# Silver decorated CeO_2_ nanoparticles for rapid photocatalytic degradation of textile rose bengal dye

**DOI:** 10.1038/s41598-020-79993-6

**Published:** 2021-01-13

**Authors:** G. Murugadoss, D. Dinesh Kumar, M. Rajesh Kumar, N. Venkatesh, P. Sakthivel

**Affiliations:** 1 Centre for Nanoscience and Nanotechnology, Sathyabama Institute of Science and Technology, Chennai, Tamil Nadu 600119 India; 2 Institute of Natural Science and Mathematics, Ural Federal University, Yekaterinburg, 620002 Russia; 3 Department of Nanoscience and Technology, Bharathiar University, Coimbatore, Tamil Nadu 641046 India

**Keywords:** Nanoscale devices, Nanoscale materials

## Abstract

High quality silver (Ag) decorated CeO_2_ nanoparticles were prepared by a facile one-step chemical method. The samples were characterized by X-ray diffraction (XRD), scanning electron microscopy (SEM), High resolution transmission electron microscopy (HR-TEM), fourier transform infrared spectrometer (FT-IR), electron paramagnetic resonance (EPR), X-ray photoelectron spectroscopy (XPS), UV–Visible absorption (UV–Vis), photoluminescence (PL) and thermogravimetric analysis. The decoration of Ag on CeO_2_ surface was confirmed by XRD, EPR and HR-TEM analysis. Harmful textile pollutant Rose Bengal dye was degraded under sunlight using the novel Ag decorated CeO_2_ catalyst. It was found that great enhancement of the degradation efficiency for Ag/CeO_2_ compared to pure CeO_2_, it can be ascribed mainly due to decrease in its band gap and charge carrier recombination rate. The Ag/CeO_2_ sample exhibited an efficient photocatalytic characteristic for degrading RB under visible light irradiation with a high degradation rate of 96% after 3 h. With the help of various characterizations, a possible degradation mechanism has been proposed which shows the effect of generation of oxygen vacancies owing to the decoration of Ag on the CeO_2_ surface.

## Introduction

Metal oxide nanoparticles have been considered in more consideration due its attractive applications including energy conversion, storage, solar fuel, photocatalytic and medical. Particularly, these metal oxides have been successfully used for wastewater management and water splitting. They have additionally been used for a wide scope of chemical redox reactions, for example, the mineralization of natural contaminations in wastewater^1,2^. The preparation of semiconductor metal oxide nanoparticles with different shapes and sizes has been of interest for application in cutting edge oxidation procedures^3,4^. Various transition metal oxides such as TiO_2_, NiO, CuO, ZnO and BiVO_4_ have been have been widely examined as photocatalysts for photocatalytic hydrogen production and color removal from the textile wastewater^5–10^. Apart from the transition metal oxides, rare earth oxides have been paid more attention due to its interesting electronic, optical and catalytic properties. As one of the most significant earth oxides, ceria (CeO_2_) has fascinated in more consideration for its promising application in photocatalytic dye degradation, solid oxide fuel cells, electrochemical sensor, ultraviolet filter, supercapacitor, solar cells and optical materials^11,12^. CeO_2_ is a n-type semiconductor metal oxide, it has a few properties like TiO_2_, for example, chemical inactivity, cheap, photo stability and non-toxicity^13–17^.

For the past one decade, color removal of organic textile dyes using suitable catalyst is an emerging target for wastewater management^18–20^. Besides, rose bengal (RB) dyes are widely used in textile, plastic, printing and cosmetic industries. But RB dyes are highly soluble in water, which commonly pollutes water and highly toxic to the living organisms^21,22^. Therefore, it is highly essential the removal of toxic dye from water. Photocatalyst is one of a most common cost-effective method for decompose textile-dyes from water using efficient nanocatalyst. To improve creation of active species in CeO_2_, various modifications were made. Among the modifications, incorporation or decoration of metal ions like silver (Ag) is an attractive method which enhanced the optical and catalytic properties of metal oxides. Such enhancement of photocatalytic behavior was obtained because of several factors such as tuning size, improved surface to volume ratio, morphology, band gap and various types of defects^23^. Dawoud et al.^24^ observed that Ag doped ZrO_2_ nanoparticles are effectively degraded the RB dye in visible light irradiation because of the surface area, band gap and porosity. Similar degradation effect has been observed by Ziashahbi et al.^25^ using Ag decorated ZnO hybrid nanostructures against methylene blue under visible light.

In this study, pure and Ag decorated CeO_2_ nanoparticles have been synthesized and characterized using experimental techniques. Crystallography, microstructure, optical and magnetic properties of the nanoparticles were systematically analysed. Photocatalytic behaviour of the pure CeO_2_ and Ag decorated CeO_2_ nanoparticles were evaluated by photodegradation of RB dye under visible-light irradiation.

## Materials and methods

### Synthesis of pure CeO_2_ and Ag decorated CeO_2_ nanoparticles

**Figure 1:**
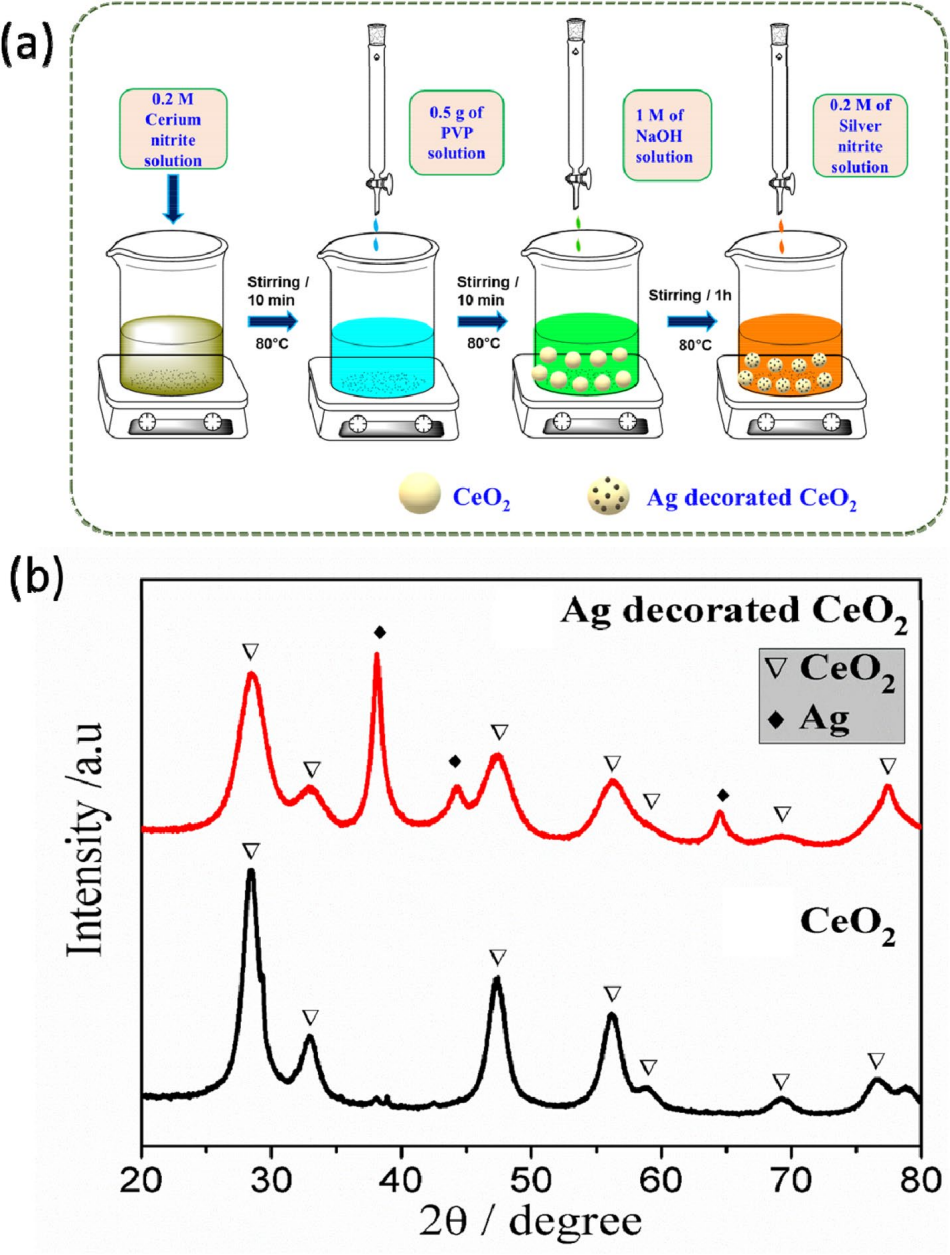
(**a**) Mechanism of the Ag decorated CeO_2_ nanoparticles preparation. (**b**) XRD spectra of as-synthesized CeO_2_ and Ag decorated CeO_2_ nanoparticles. The asterisks (•) and (∆) are represents corresponds to pure CeO_2_ and Ag and CeO_2_ characteristic diffraction peaks.

All chemicals were used without further purification with analytical grade reagent. To synthesis of Ag doped CeO_2_ nanoparticles, 4.3 g (0.2 M) of Ce (NO_3_)_3_·6H_2_O in 50 mL of deionized water and 50 ml of 0.5 g polyvinylpyrrolidone (PVP; MW:40,000) were mixed under stirring at 80 °C. Then, 1 M of sodium hydroxide (NaOH) was added drop by drop into the above solution. After 1 h, 0.2 M of 1.7 g Ag(NO_3_) in 50 mL deionized water was added into the above solution. Subsequently, that the white color solution was transformed into dark brown color solution indicates the decoration of Ag on the surface CeO_2_ nanoparticles. After 2 h stirring, the obtained brown color colloidal was purified by washing with deionized water and acetone with several times to remove impurities. The resultant powder samples were dried in an oven at 120 °C for 4 h. Following the same procedure, pure CeO_2_ nanoparticles was synthesized without adding the silver nitrate. The growth mechanism of Ag decorated CeO_2_ is demonstrated in Fig. 1a.

### Characterization

Structural information of the pure and doped CeO_2_ was studied by powder X-ray diffraction (XRD) pattern using Rigaku miniFlex IIC diffractometer with Cu Kα radiation (λ = 1.54060 Å). Morphology of the powder samples and microstructural information were performed using scanning electron microscopy (SEM, TESCAN, VEGASEM) and HR‒TEM (JEOL 3010) microscopy. Functional groups of molecules presented in the surface of the samples were investigated by FT-IR using an AVATOR 360 spectrometer. Chemical state of the compounds studied using X-ray photoelectron spectroscopy with an Omicron Nanotechnology The chemical state in the nanocomposites was investigated by X-ray photoelectron spectrometer (XPS) using an ESCA + Omicron XPS system with a Mg-Kα source and photon energy of 1486.7 eV. Free radical information of the samples was studied by Electron paramagnetic spectroscopy (EPR) using Bruker EMX Plus Electron spectrometer. Optical properties of the samples were investigated using Varian Carry 5000 scan UV–Vis double beam spectrometer. Thermal stability and phase transition of the sample was evaluated by thermogravimetric and differential thermal analysis (TG–DTA) using DST Q600 20 thermometer with heating rate of 10°/min.

### Photocatalytic study

Photodegradation of rose bengal dye was studied using pure CeO_2_ and Ag decorated CeO_2_ nanoparticles in aqueous medium under sun light. For the photocatalytic reaction, 20 mg of photocatalyst was dispersed into 50 mL rose bengal dye solution, the concentration of dye was used as 0.2 g/L. The resultant solution was stirred in dark place for 1 h before the light irradiation. Then the solution was irradiated for 3 h under sun light. To evaluate degradation performance, the irradiated solution takes out in certain time interval and it was centrifuged at 4000 rpm for 20 min. Finally, the degradation efficiency of the reacted solution was determined using UV–Vis absorption spectroscopy. The degradation study was performed under 1180 watts per square meter light intensity. The light intensity was measured using the flux meter.

## Results and discussion

### Structural properties of CeO_2_ and Ag-CeO_2_ nanoparticles

The XRD patterns of pure and Ag decorated CeO_2_ nanoparticles are shown in Fig. 1b. The pure nanoparticles show well–defined cubic structure of CeO_2_ (Fig. 1b) with the characteristic plane of (1 1 1) orientation. The obtained result is well matched with standard JCPDS data (file no. 34-0394). In the case of Ag incorporated CeO_2_ nanoparticles, crystalline Ag peaks are obtained along CeO_2_ peaks with the primary characteristic of Ag (1 1 1) plane. The attained Ag peaks are well matched with cubic structure of Ag (JCPDS file no. 87-0717). Moreover, there is no obvious peak shifts observed in CeO_2_ pattern by addition of Ag which reveals that Ag is presented on surface rather occupied in the interstitial or vacancy sites of CeO_2_. The estimated average crystallite size of pure CeO_2_ nanoparticle is 8.1 ± 1 nm and the size decreased to 6.5 ± 1 nm for the Ag incorporated CeO_2_ nanoparticles. The decrease in crystallite size is clearly evident from the peak broadening as shown in Fig. 1b. Besides the size of Ag crystallites found to be ~ 7.9 nm. The obtained predominant Ag peaks are revealed that equally distributed on the CeO_2_ surface. Moreover, it can be noticed that the calculated lattice parameter (~ 5.414 Å) of as-synthesized CeO_2_ nanoparticle is slightly higher than that of bulk CeO_2_ (~ 5.411 Å). For Ag incorporated samples, a higher value of CeO_2_ lattice parameter (~ 5.553 Å) is obtained which may be due to higher surface defects and decreasing crystallite size.

### Microstructural analysis of pure and Ag incorporated CeO_2_ nanoparticles

**Figure 2:**
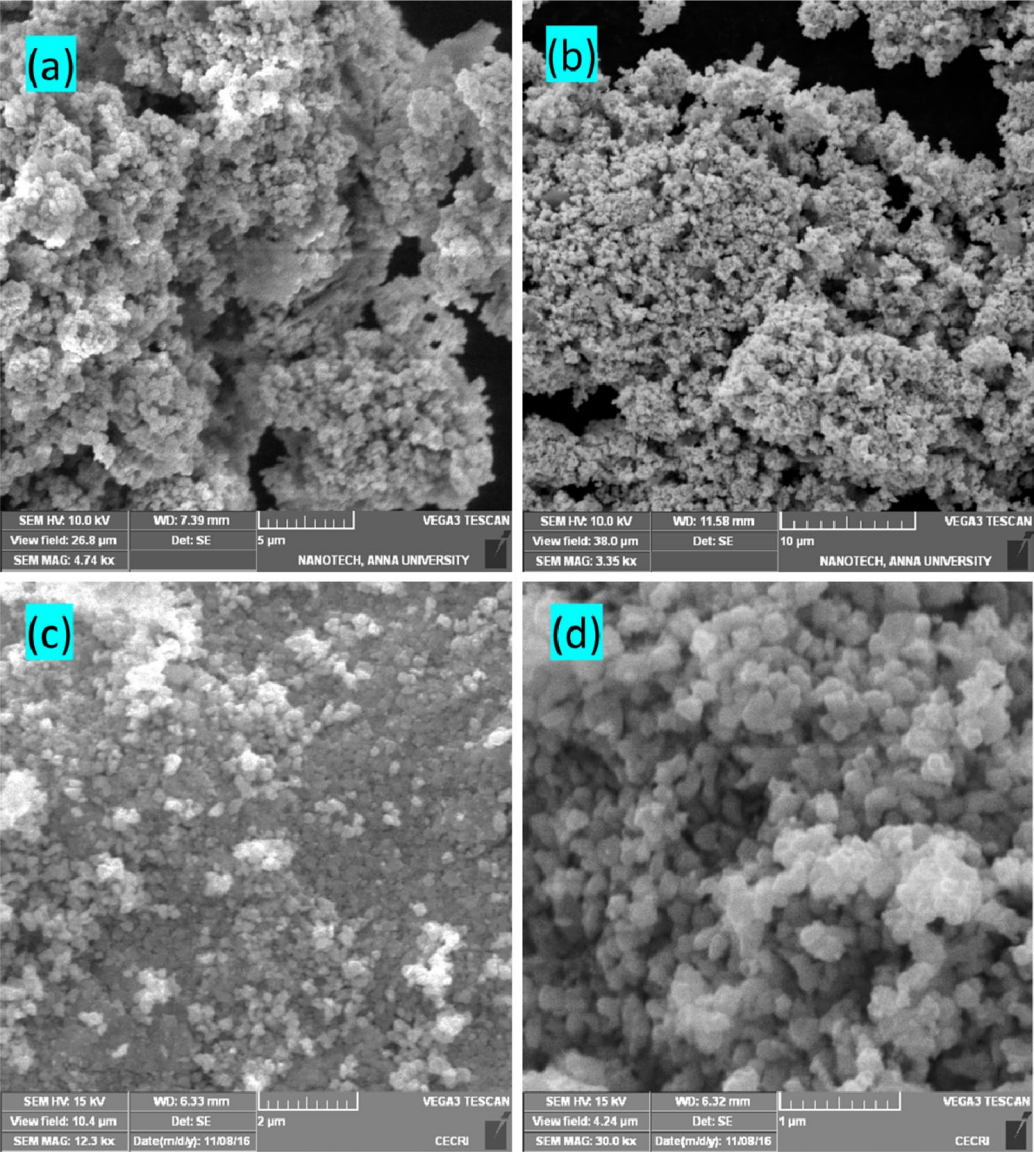
Morphological analysis of (**a**,**b**) pure CeO_2_ and (**c**,**d**) Ag decorated CeO_2_ nanoparticles.

**Figure 3:**
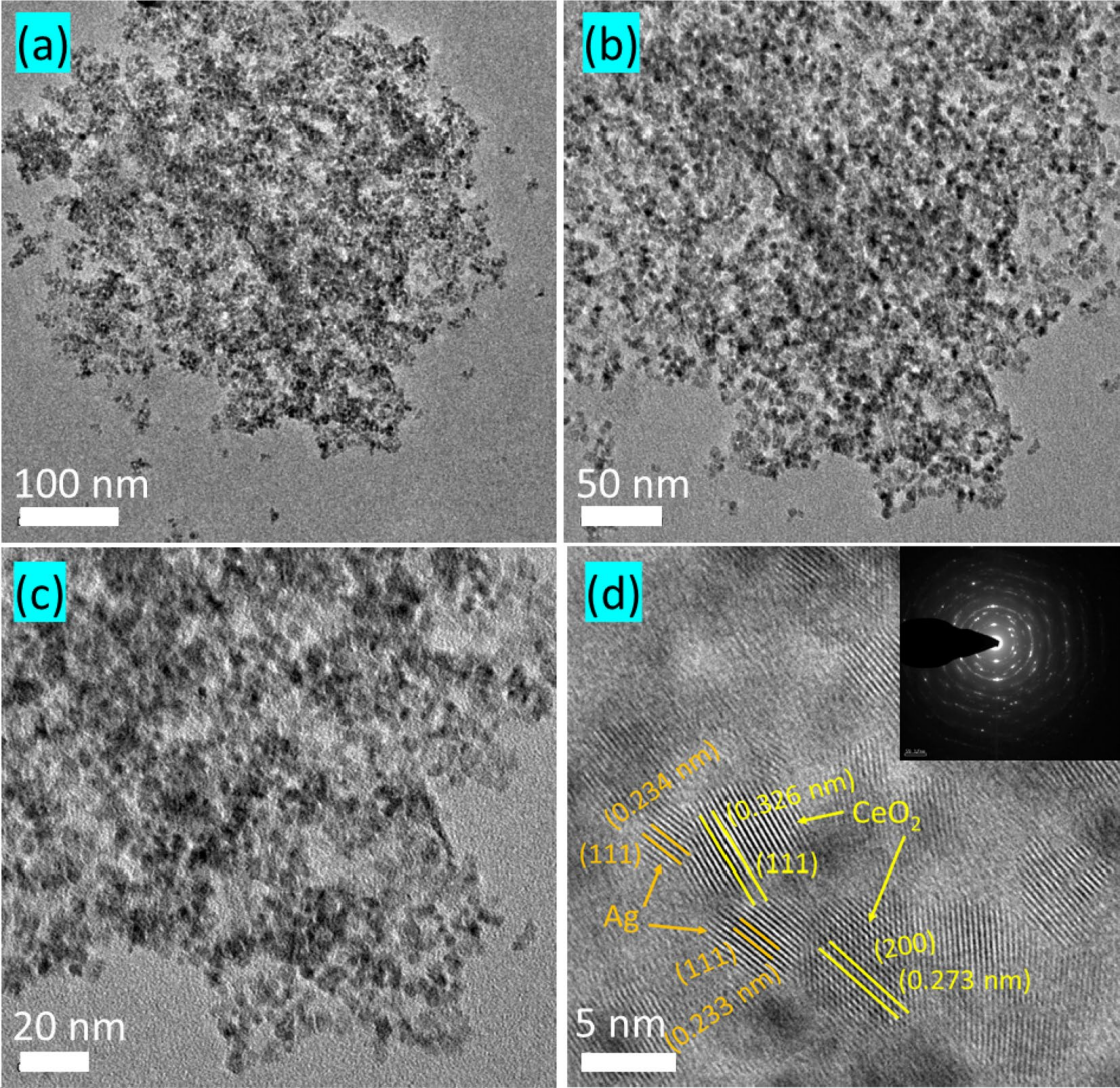
(**a**–**c**) HR-TEM analysis of Ag decorated CeO_2_ nanoparticles with different magnifications. (**d**) Shows high magnification of HR-TEM images with well-resolved lattice springs and SAED pattern (insert) of Ag decorated CeO_2_ nanoparticles.

Microstructural characteristics of pure and Ag decorated CeO_2_ nanoparticles are examined using SEM technique with different magnifications and the results are presented in Fig. 2a–d. It can be clearly seen that the obtained particle sizes are obviously in nanoscale range with agglomeration. Besides, pure CeO_2_ nanoparticles are spherical in shape (Fig. 2a,b), whereas, the Ag decorated nanoparticles are relatively increasing size and defined grain boundaries as seen in Fig. 2c,d. This microstructure result implies that the crystalline Ag might be surrounded over the CeO_2_ particles. The formation of two distinct crystalline phases (Ag and CeO_2_) is evident from the XRD results. To further investigate this microstructure, the Ag decorated sample is investigated using HR-TEM analysis. Figure 3a–d shows the different magnification images of Ag decorated nanoparticles, it showed uniform size and shape with homogenous distribution. The higher magnification results clearly have shown the crystallites with size ranges between 6 and 8 nm. The estimated d spacing between the lattice fringes about 0.326 nm and 0.233 nm corresponding to the respective (1 1 1) plane of cubic structure of Ag and CeO_2_, respectively. The estimated grain size and structure in SAED patterns (insert Fig. 3d) are in good agreement with the XRD results. The distinguish fringes in SAED pattern is indicates we crystalline nature of the sample.

### FT-IR study

**Figure 4:**
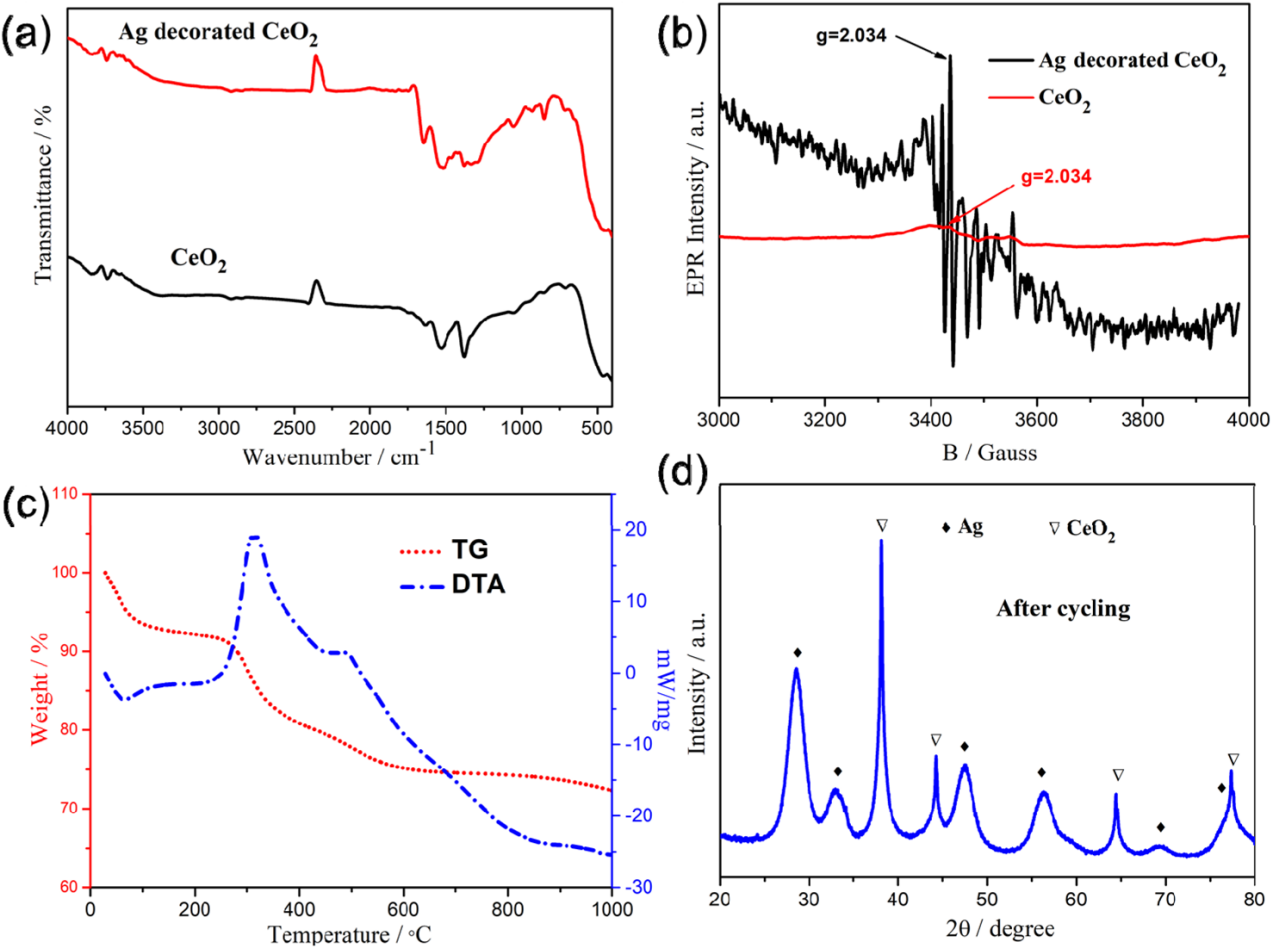
(**a**) FT-IR spectra of pure CeO_2_ and Ag decorated CeO_2_ nanoparticles, (**b**) EPR spectra of pure CeO_2_ and Ag decorated CeO_2_ nanoparticles, (**c**) thermal analysis (TG/DTA) of Ag decorated CeO_2_ nanoparticles, and (**d**) XRD of Ag decorated CeO_2_ nanoparticles after cycling test.

Figure 4a illustrates the FT-IR spectra to analyse the adsorption species on the surfaces of as-synthesized CeO_2_ and Ag decorated CeO_2_ nanoparticles. The major absorption band observed at 460 cm^−1^ is due to the deformation mode of Ce–O bond. The other peaks obtained at 1389 cm^−1^ corresponding to the O–H bending vibrations and the peak at 1010 cm^−1^ due to the stretching vibrations of Ce–O. It can be clearly noticed that the Ag decorated CeO_2_ samples shows additional peak at 800 cm^−1^ which could be due to the formation of Ag–O stretching vibrations (non-bridging). The peak around 1600 cm^−1^ can be attributed to the symmetric bending of H_2_O and the peak around 2350 cm^−1^ divulges the stretching vibrations of C-O adsorbed from the atmospheric CO_2_^24^. The minor peak at 3400 cm^−1^ could be observed owing to the stretching vibration of O–H group.

### EPR studies

Electron paramagnetic resonance (EPR) spectroscopy is a more sensitive technique to analysis about doping process or surface effect, particularly evaluate electronic configurations with unpaired spins. Figure 4b shows EPR spectra for pure and Ag decorated CeO_2_ nanoparticles. The obtained result showed presence of paramagnetic species. Perceptible the signal showed between 3300 and 3600 G for Ag incorporated CeO_2_ demonstrates Ce^3+^ defect states. The obvious spectral changes (Fig. 4b) are clearly showing possibility of defects enhancement by decoration of Ag with CeO_2_ surface. To pure CeO_2_, it is possible to develop ∙OH free radical through Ce^3+^/Ce^4+^ redox cycle by activation of the water molecules on the oxygen vacancy site. The developed oxygen vacancy can boost degradation efficiency. In the case of Ag decorated CeO_2_, the photocatalytic activity could further increase by electron acceptor (Ag^2+^ ↔ Ag^0^) and/or hole donor (Ag^2+^ ↔ Ag^+^) along with concentration of ceria redox and oxygen vacancies^26^. The strong EPR signals are all attributed to the O^2−^ species on the CeO_2_ surface. The obtained highest photocatalytic activity caused by the highest surface oxygen vacancy concentration, as well as existence of lattice oxygen species and lattice defects formed with the participation of both silver and ceria. The multiple spectral lines in the EPR spectrum for Ag decorated CeO_2_ sample shows possibility of the formation of acceptor and donor during light irradiation. Hence, the obtained EPR revealed that the Ag decorated CeO_2_ nanoparticles can be performing as a good photocatalyst due to creation of more free radicals.

### Thermal study

The thermal analysis of Ag decorated CeO_2_ nanoparticles are carried out up to 1000 °C and the resultant curves are shown in Fig. 4c. The TG results indicate the total weight loss ~ 28% up to 1000 °C. It can be clearly seen that the continuous weight loss has occurred up to ~ 550 °C. With further increasing the temperature up to 900 °C, drastic weight loss observed indicates the complete dehydration. But much higher temperature (> 900 °C), the weight loss started gradual increase in trend as shown in Fig. 4c. This is due to the temperature reached the melting point of Ag (~ 960 °C) followed by the silver decomposition takes place. The DTA plot illustrates broad peak appeared up to 250 °C related to the decomposition and elimination of water and other adsorbed molecules. In addition, the strong exothermic peaks between 250 and 550 °C are attributed to the oxidation and crystallization takes place in the nanoparticles. It has been reported that the crystallization of Ag takes place at the temperature from 200 to 300 °C^27^. From the TG and DTA results, it can be established that the Ag decorated CeO_2_ nanoparticles are highly thermal stable upto 250 °C. To confirm the stability of the catalyst, XRD spectrum was recorded after 5 cycles of photodegradation experiment. Figure 4d shows XRD spectrum of Ag decorated CeO_2_ nanoparticles after cycling test. It clearly shows the diffraction planes in the Fig. 4d are identical with before cycling sample (Fig. 1b), it indicates the sample is more stable even after 5 cycling tests.

### X-ray photoelectron spectroscopy (XPS)

**Figure 5:**
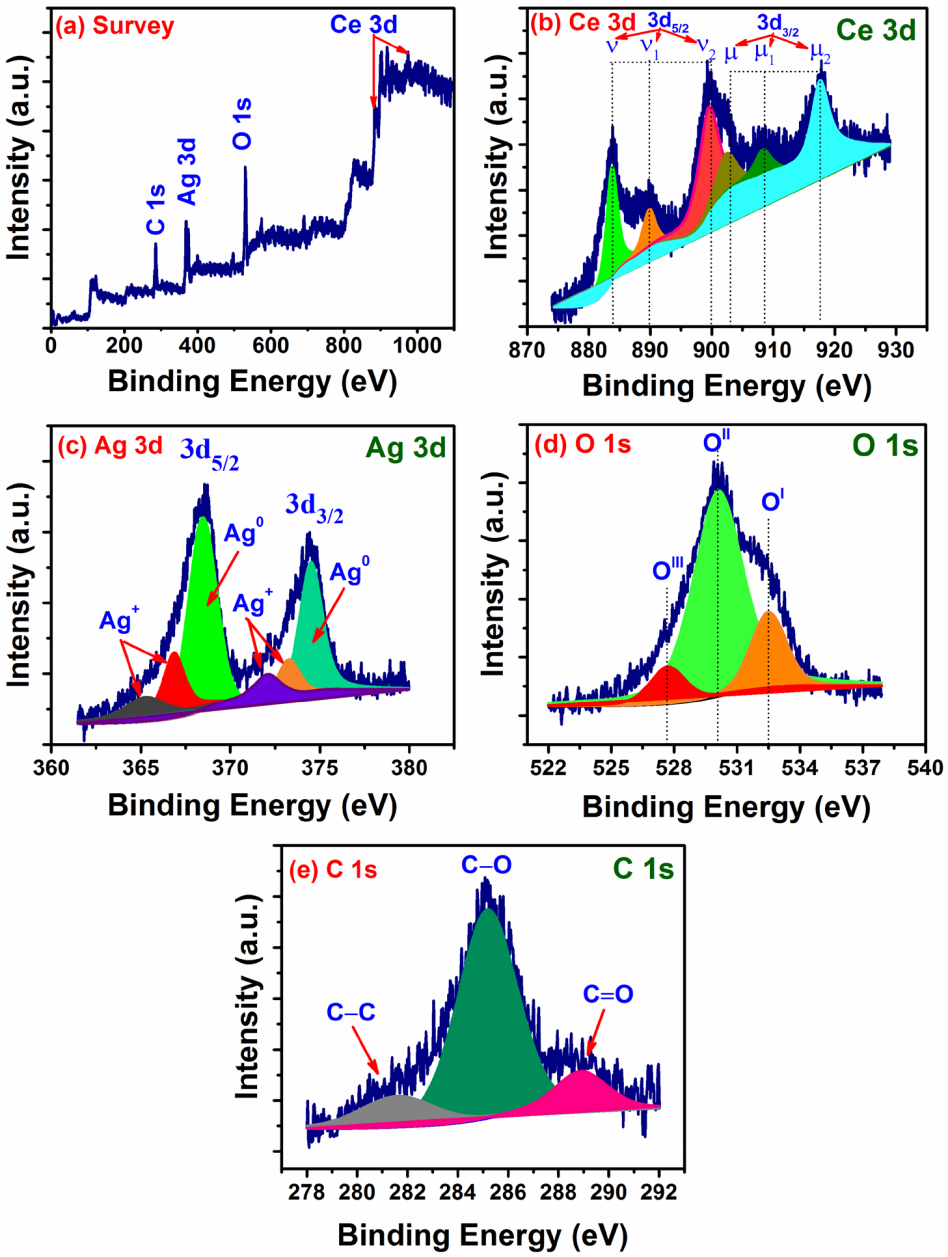
XPS survey and high-resolution spectra of Ag doped CeO_2_ nanoparticles. (**a**) Survey, (**b**) Ce 3d, (**c**) Ag 3d, (**d**) O 1 s and (**e**) C 1s.

Chemical bonding nature of Ag decorated CeO_2_ nanoparticles is analysed using XPS technique. The survey spectrum in Fig. 5a indicates the sample is mainly composed of Ce, O and Ag with the respective binding energies of 880–920 eV (Ce 3d), ~ 530 eV (O 1s) and ~ 360 eV (Ag 3d). Figure 5b divulges the high-resolution Ce 3d spectra consist of spin–orbit doublets Ce 3d_5/2_ (~ 882 eV) and Ce 3d_3/2_ (917 eV). These binding energies are well-assigned to the predominant Ce^4+^ and Ce^3+^ oxidation states of CeO_2_^28^. The Ce 3d region has well-separated spin–orbit splitting about 18.6 eV. Besides, it can be seen that the satellite peaks (ν_1_, ν_2_, μ_1_ and μ_2_) are observed along with Ce 3d_5/2_ and Ce 3d_3/2_ peaks in Fig. 5b related to the energy-gain (shake-down) process^29^. On the other hand, signature of metallic Ag peaks is observed in the Ag 3d core-level spectra as shown in Fig. 5c. The peaks at 368.2 eV (Ag 3d_5/2_) and 374.3 eV (Ag 3d_3/2_) are metallic Ag (Ag^0^) and well-separated spin–orbit coupling of ~ 6 eV. The obtained results are well-agreement with the previous report^30^. The high intensity of Ag 3d peaks due to the presence of higher Ag concentration (> 10 wt%) in the nanoparticles which corroborates with the XRD results. Besides, the satellite peaks around the lower binding energy sides of Ag 3d_5/2_ (~ 365.1 eV and ~ 366.8 eV) and Ag 3d_3/2_ (~ 372.1 eV and ~ 373.2 eV) are attributed to the Ag^+^ oxidation states. The deconvoluted O1s (Fig. 5d) spectra indicates the three major binding energies 527.8 eV (O^III^), ~ 530 eV (O^II^) and ~ 532.5 eV (O^I^) are associated to the lattice oxygen ions (O_lat_) and surface adsorbed oxygen ions (O_ads_). The signature of surface adsorbed oxygen could be observed due to the oxygen vacancies in the crystal structure resulting from the enrichment of Ag ions^29,30^. In addition, C 1S peak (Fig. 5(e)) is observed corresponding to the binding energies of ~ 281.5 eV (C–C), ~ 285.1 eV (C–O) and ~ 288.9 eV (C=O) indicates the presence of organic carbon in the nanoparticles. Interestingly, no hydroxide peaks are detected in the Ag decorated CeO_2_ nanoparticles.

**Figure 6:**
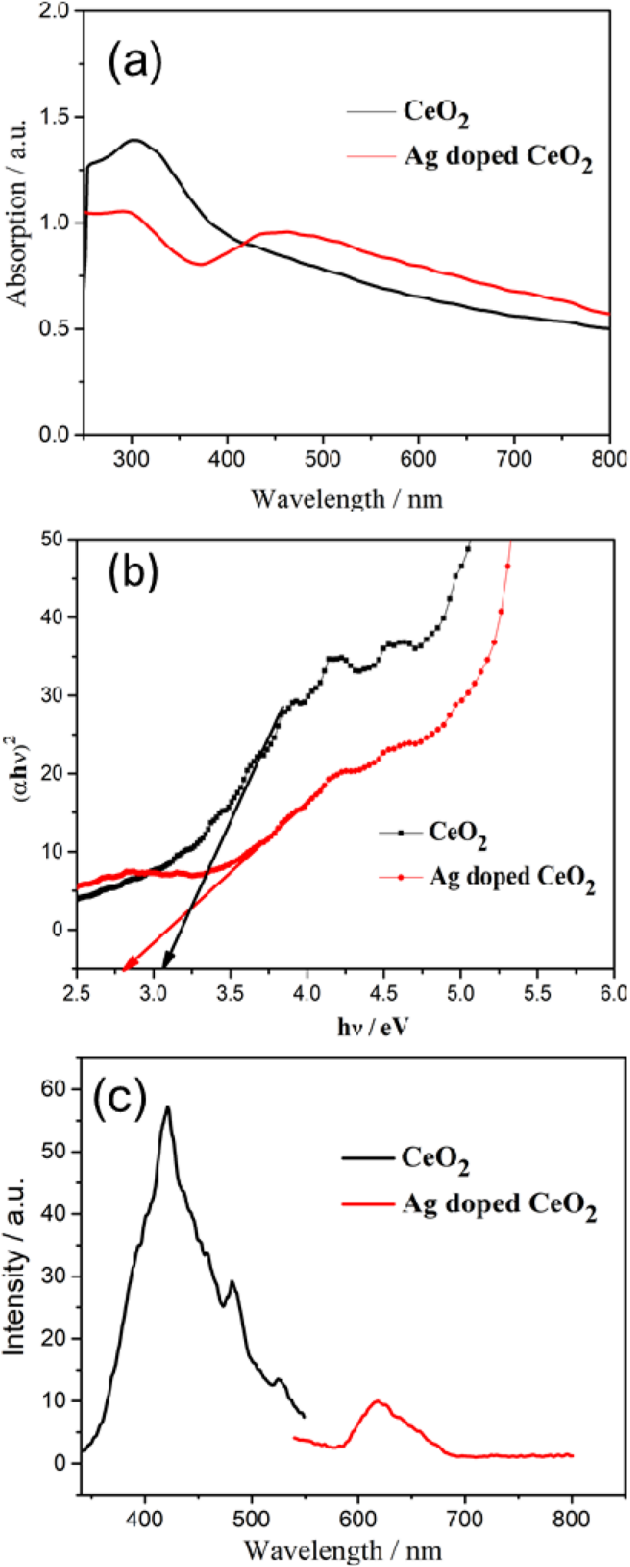
Optical absorption of pure CeO_2_ and Ag decorated CeO_2_ nanoparticles, (**a**) UV–Vis spectra, (**b**) Tauc plot and (**c**) PL spectra.

Figure 6a–c demonstrates the UV–visible and florescence spectra of pure CeO_2_ and Ag decorated CeO_2_. A strong absorption peak obtained around 320 nm for the pure CeO_2_ nanoparticles, and this peak is shifted towards higher wavenumber at 430 nm for the Ag decorated samples, as shown in Fig. 6a. This peak variation depicts the defects in the form of oxygen vacancies induced by the higher amount of Ag ions around the CeO_2_ particles. Tauc plot (Fig. 6b) illustrates the variation in band gap energy of the nanoparticles. The band gap of pure CeO_2_ is found to be 3.1 eV and the band gap increased to 2.8 eV for the Ag decorated CeO_2_ nanoparticles. The photoluminescence emission spectra of pure CeO_2_ and Ag decorated CeO_2_ nanoparticles are shown in Fig. 6c using an excitation wavelength of 365 nm. For pure CeO_2_ nanoparticles, the spectrum showed an intense emission peak in blue region 425 nm corresponding to the surface related defects and the other minor shoulder peaks at 460 nm and 480 nm are attributed to the dislocation or oxygen vacancies. In addition, emission at 535 nm could be anticipated from the oxygen vacancies associated defects^31^. In the case of Ag decorated CeO_2_ nanoparticles, the emission were observed in red region centred at 615 nm with much lower intensity as compared to the pure CeO_2_ nanoparticles. This red region emission are expected from the higher surface defects by the incorporation of more Ag metal ions as already revealed in microstructural analysis. Depending on the concentration of surface defects, the recombination process of electrons and holes are delayed which results lower intensity than pure CeO_2_. It has been reported that the delayed recombination process facilitates the large number of photo-generated electrons and holes during photochemical reactions^32^. Such behaviour is beneficial for the enhanced photocatalytic activity of Ag decorated CeO_2_ nanoparticles.

### Photocatalytic studies

The photocatalytic properties studied for the prepared pure CeO_2_ and Ag decorated CeO_2_ samples, the degradation of Rose Bengal (RB) used as a model dye by irradiation of visible sun light. Especially, the activity of prepared photocatalysts were depends on important factors like crystal structure, size of catalytic particle morphology and composition concentration. The efficiency of photocatalytic degradation premeditated by the decolourisation of model pollutant was determined by UV–Visible spectra.

**Figure 7:**
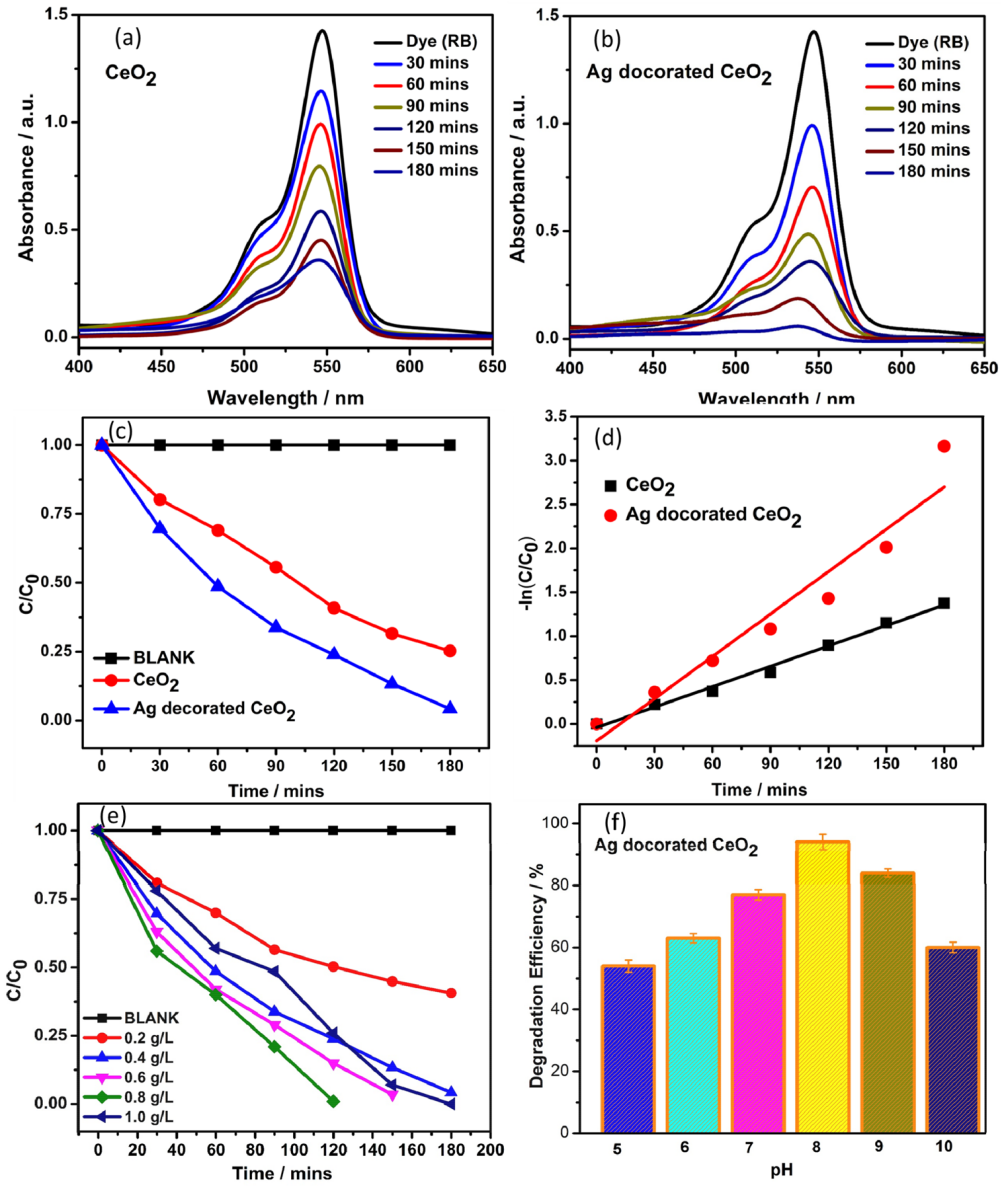
(**a**,**b**) UV–Vis spectra of the CeO_2_ and Ag decorated CeO_2_ catalyst dispersed in Rose bengal dye dispersed solution with light irradiation of different time interval, respectively. (**c**,**d**) (C/C_0_) versus time interval and Plot of − ln(C/C_0_) versus time interval, respectively. (**e**) Photocatalytic degradation of rose bengal at different catalytic loads (RB—20 ppm pH-8). (**f**) Effect of PH on 50 mL of 20 ppm with 20 mg Ag decorated CeO_2_ catalyst.

Figure 7a,b represents the UV–Visible spectra of RB dye degradation using CeO_2_ and Ag decorated CeO_2_ catalyst with irradiation time interval of 180 min under sun light. The absorption of dye molecule is gradually reduced with intensifying irradiation time of degradation due to deprivation of chromophore to form a transitional product. The degradation efficiency was calculated by,

1 $${\text{Degradation }}\% \, = \, \left\{ {\left( {{\text{C}}_{0} {-}{\text{C}}} \right)/{\text{C}}_{0} } \right\} \, \times { 1}00$$

where C_0_ and C are the initial and final concentration of aqueous dye solution (20 mg/L). The degradation study was carryout with different catalysts such as CeO_2_ and Ag decorated CeO_2_ catalyst. The degradation efficiency was calculated using the Eq. (1). The obtained degradation efficiency is presented in Fig. 7c.

The CeO_2_ catalyst achieved the degradation efficiency of about 74% against RB under the irradiation of visible sun light for 180 min. However, the Ag decorated CeO_2_ catalyst, the degradation was conducted by the same process interestingly the degradation rate was dramatically increased. The maximum efficiency obtained by the catalyst is 96% (Ag decorated CeO_2_) is significantly higher efficiency when compared with CeO_2_ due to the energy level of the Ag acts as trapper and promote the higher and easier reactive oxygen species generation. Particularly, the photo generated electron in the conduction band is effectively separated and then migrated with the energy levels of the Ag leads to reduction process. Simultaneously, in the valence band holes catalyst leads to that holes that reacts with H_2_O molecules that leads to the formation hydroxyl ions then promotes to hydroxyl radicals under the irradiation of visible sun light. Plasmonic effect is an another major reason to enhancing the photocatalytic performance of Ag decorated CeO_2_ under visible light^33–35^.

The Lagergren rate equation is widely used rate equation for the degradation of adsorbate from aqueous solution. The Lagergren first-order model can be represented as

2 $$K \, = - \, \ln \, C/C_{0} ,$$

where K was kinetic constant and the kinetic rate constants are studied by plotting—ln(C/C_0_) with the irradiation time (min), which is depicted in Fig. 7d. The results obtained from the pseudo first-order kinetic model beside with the experimental k and R^2^ values are presented in Table 1.

**Table 1:** Photocatalytic activity studies of CeO_2_ and Ag decorated CeO_2_ nanoparticles.

S. no	Catalyst	Band gap (eV)	Degradation efficiency (180 min, 20 ppm of RB)	K (min^−1^)	R^2^
1	CeO_2_	3.1	74.6	0.0178	0.9536
2	Ag/CeO_2_	2.8	95.7	0.0370	0.9262

In Fig. 7d, the kinetic constants (k) calculated from the photocatalytic degradation of CeO_2_ and Ag decorated CeO_2_ catalysts were 0.0178, and 0.0370 min^−1^, respectively. The kinetic constants showed the Ag decorated CeO_2_ catalyst had superior performance for RB dye photocatalytic degradation under the sun light. The effect of catalyst loads of Ag decorated CeO_2_ nanoparticles for photocatalytic degradation was calculated 50 mL of 20 ppm dye solution at pH 8. As we know that the amount catalyst influences the degradation rate. Especially in this catalytic dosage enhance the ROS generation and increase the overall efficiency but after certain concentration the turbidity of the solution increases due to inter collisions between the particles, it becomes block the penetration of radiation. So overall efficiency again decreased. Figure 7e is clearly indicating the optimum dose value of 0.8 g/L is showed higher efficiency of photocatalytic degradation.

The pH of the solution also affects the photo degradation efficiency. The effect of pH on the photocatalytic degradation of dye was investigated by 50 mL of the dye solution with 10 mg of Ag decorated CeO_2_ nanoparticles and the pH value was varied from 5 to 10 by adding of 0.1 N NaOH and HCl. The surface charge of catalyst is altered imparts a change in the overall efficiency. Under the lower pH value, the anionic dye in their protonated form and catalyst also owns positive charge due to adsorption of H^+^ ions. So, in the acidic medium, the dye molecule repel the from the catalyst and shows lower degradation rate. As seen in the Fig. 7f, when the pH is increased from 5 to 10, initially increased the degradation rate upto pH 8, after that it was decreased. At the higher pH solution, the dye molecule and catalyst interaction increased resulting increasing degradation efficiency. Further increasing the pH value more than 8, the surface of catalyst becomes negatively charged and it promote the repulsion of dye molecule due to that reduced the overall efficiency^36,37^. Table 2 shows comparison of CeO_2_ and Ag doped CeO_2_ of present work with previously reported photocatalysts.

**Table 2:** Comparison of current and reported studies of various CeO_2_ based nanoparticles.

Catalyst	Dye	Dye concentration	Catalyst concentration	Light source	Time duration (min)	Efficiency (%)	Ref
Ag/CeO_2_	MB	20 ppm	50 mg	Visible	30	95	^38^
Ag/CeO_2_	MB	10 ppm	50 mg	Visible	150	100	^39^
Sm/CeO_2_	RB	5 ppm	50 mg	Visible	90	90	^29^
Ag/CeO_2_	RhB	5 ppm	50 mg	Visible	70	100	^40^
Ag/CeO_2_	MB	5 ppm	50 mg	Visible	60	97	^40^
Ag/CeO_2_	CV	5 ppm	50 mg	Visible	60	99	^40^
CeO_2_/V_2_O_5_	MB	10 ppm	20 mg	Visible	25	98	^41^
Ag/CeO_2_	MO	16 ppm	10 mg	Sunlight	60	84	^42^
CeO_2_-BiVO_4_/FAC	MB	10 ppm	200 mg	Visible	180	90	^43^
Au@CeO_2_	MB	10 ppm	2 mg	Visible	300	95	^44^
Au@CeO_2_	MO	10 ppm	2 mg	Visible	360	80	^44^
Bi_2_O_3_/CeO_2_	RhB	10 ppm	100 mg	Visible	200	74	^45^
Fe@CeO_2_	MB	0.5 mM	1 × 1 film	Visible	180	80	^46^
CeO_2_ hollow sphere	RhB	20 ppm	10 mg	Visible	180	93	^47^
Ag decorated CeO_2_	RB	20 ppm	10 mg	Sunlight	180	96	Present work

**Figure 8:**
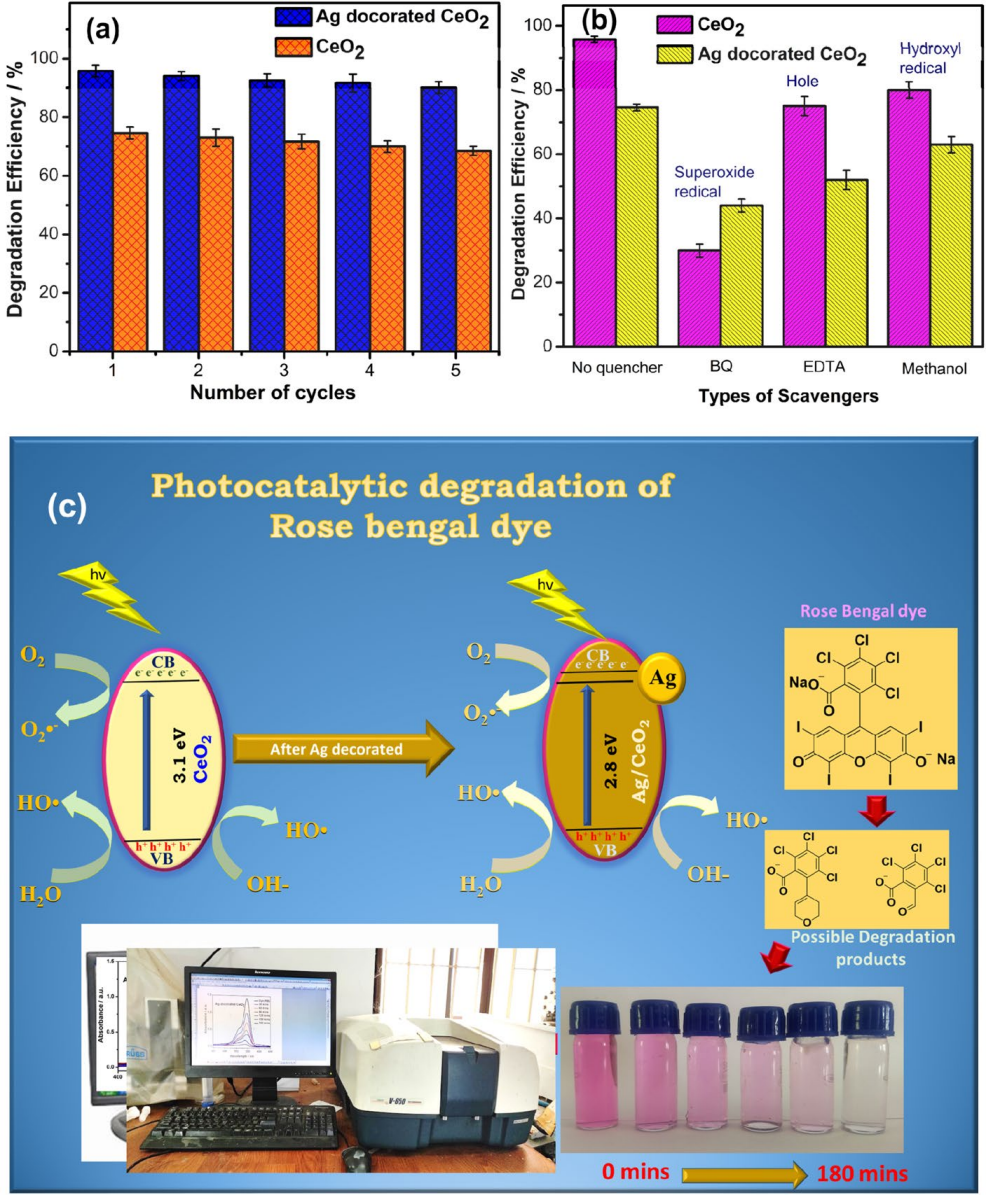
(**a**) Stability test of the CeO_2_ and Ag decorated CeO_2_ catalyst after 5 cycles. (**b**) Scavenger studies of CeO_2_ and Ag decorated CeO_2_ catalyst. (**c**) Schematic diagram illustrating the mechanism of charge separation and photocatalytic activity of photocatalyst under the sunlight irradiation.

Stability test of the CeO_2_ and Ag decorated CeO_2_ catalyst is showed as 68.5% and 90%for RB dye after 5 cycles as shown in Fig. 8a. Furthermore, the presence of Ag accelerates the charge separation and reactive oxygen species (ROS) generation. The study of scavenger test is helpful to understand the mechanism of the photodegradation of dyes over active photocatalyst. Hence, the h^+^, OH^−^, and ^.^O_2_^−^ are eliminated by adding EDTA (h^+^ scavenger), methanol (·OH scavenger), and p-BQ (·O_2_^−^ scavenger) into reaction solution. Figure 8b depicts the efficiency of with and without scavengers studies. The results reveal the influence of super oxide radicals because, addition of Benzoquinone shows less efficiency. The photocatalytic mechanism of CeO_2_ and Ag decorated CeO_2_ is shown in Fig. 8c. The mechanism of MB degradation is described as follows,

3 $${\text{CeO}}_{{2}} ,{\text{ Ag}}/{\text{CeO}}_{{2}} + {\text{ h}}\nu \to {\text{h}}^{ + } \left( {{\text{VB}}} \right) \, + {\text{ e}}^{-} \left( {{\text{CB}}} \right),$$

4 $${\text{OH}}^{-} + {\text{ h}}^{ + } \to {\text{OH}}\cdot,$$

5 $${\text{H}}_{{2}} {\text{O }} + {\text{ h}}^{ + } \to {\text{OH}}\cdot \, + {\text{ H}}^{ + } ,$$

6 $${\text{O}}_{{2}} + {\text{ e}}^{-} \to {\text{O}}_{{2}}^{{ \cdot {-}}} ,$$

7 $${\text{Dye }} + {\text{ OH}}\cdot \, + {\text{ O}}_{{2}}^{{ \cdot {-}}} \to {\text{Degradation products}}{.}$$

## Conclusions

In summary, the pure and Ag/CeO_2_ nanoparticles were synthesized and characterized the structural, morphological and optical properties using various characterization techniques. The presence of Ag on CeO_2_ surface was confirmed by XRD, XPS and EPR spectroscopy. The UV absorption peak of pure and Ag decorated CeO_2_ was found at 320 nm and 430 nm and corresponding band gap calculated as 3.1 eV and 2.8 eV, respectively. The enhanced catalytic performance of Ag/CeO_2_ composites strongly depends on the preparation method that determines the homogeneous morphology and equally distribution of Ag on the ceria surface. Moreover, the obtained highest photocatalytic activity has proposed due to the presence of the formation of optimum level of Ce^3+^, oxygen vacancies and plasmonic effect. The obtained remarkable degradation efficiency for Ag decorated CeO_2_ nanoparticles promotes its utilization in environmental remediation. We believe that this work would have a considerable impact on the future development of efficient plasmonic metal–semiconductor photocatalysts.
